# Kinetic Evaluation of the Hypoxia Radiotracers [^18^F]FMISO and [^18^F]FAZA in Dogs with Spontaneous Tumors Using Dynamic PET/CT Imaging

**DOI:** 10.1007/s13139-022-00780-4

**Published:** 2022-10-11

**Authors:** Sangkyung Choen, Michael S. Kent, Abhijit J. Chaudhari, Simon R. Cherry, Ana Krtolica, Allison L. Zwingenberger

**Affiliations:** 1 Department of Surgical and Radiological Sciences, School of Veterinary Medicine, University of California, Davis, CA USA; 2 Department of Radiology, School of Medicine, University of California, CA Sacramento, USA; 3 Department of Biomedical Engineering, College of Engineering, University of California, Davis, CA USA; 4 Omniox Inc, Palo Alto, CA USA

**Keywords:** Nuclear scintigraphy, Hypoxia, Companion animal, Neoplasia, Kinetic modeling

## Abstract

**Purpose:**

We evaluated the kinetics of the hypoxia PET radiotracers, [18F]fluoromisonidazole ([18F]FMISO) and [18F]fluoroazomycin-arabinoside ([18F]FAZA), for tumor hypoxia detection and to assess the correlation of hypoxic kinetic parameters with static imaging measures in canine spontaneous tumors.

**Methods:**

Sixteen dogs with spontaneous tumors underwent a 150-min dynamic PET scan using either [18F]FMISO or [18F]FAZA. The maximum tumor-to-muscle ratio (TMR_max_) > 1.4 on the last image frame was used as the standard threshold to determine tumor hypoxia. The tumor time-activity curves were analyzed using irreversible and reversible two-tissue compartment models and graphical methods. TMR_max_ was compared with radiotracer trapping rate (*k*_3_), influx rate (*K*_i_), and distribution volume (*V*_T_).

**Results:**

Tumor hypoxia was detected in 7/8 tumors in the [18F]FMISO group and 4/8 tumors in the [18F]FAZA group. All hypoxic tumors were detected at > 120 min with [18F]FMISO and at > 60 min with [18F]FAZA. [18F]FAZA showed better fit with the reversible model. TMR_max_ was strongly correlated with the irreversible parameters (*k*_3_ and *K*_i_) for [18F]FMISO at > 90 min and with the reversible parameter (*V*_T_) for [18F]FAZA at > 120 min.

**Conclusions:**

Our results showed that [18F]FAZA provided a promising alternative radiotracer to [18F]FMISO with detecting the presence of tumor hypoxia at an earlier time (60 min), consistent with its favorable faster kinetics. The strong correlation between TMR_max_ over the 90–150 min and 120–150 min timeframes with [18F]FMISO and [18F]FAZA, respectively, with kinetic parameters associated with tumor hypoxia for each radiotracer, suggests that a static scan measurement (TMR_max_) is a good alternative to quantify tumor hypoxia.

**Supplementary Information:**

The online version contains supplementary material available at 10.1007/s13139-022-00780-4.

## Introduction

Tissue hypoxia develops when oxygen supply to tissues does not adequately meet cellular demands [1]. Tumor hypoxia has been associated with a poor prognosis, an aggressive phenotype, resistance to radiotherapy and chemotherapy, and increased risk of invasion and metastasis [1]. Therefore, knowledge of the extent and location of tumor hypoxia could allow clinicians to pursue more robust treatment approaches to counterbalance the resistance to chemo- and radio-therapy, potentially leading to improved therapeutic outcome [2]. It can also enhance development of therapeutic approaches that specifically target tumor hypoxia.

Positron emission tomography (PET) imaging with hypoxia-specific radiotracers can provide noninvasive, 3-dimensional quantitative assessment of intratumor oxygen levels, is repeatable during treatment enabling monitoring of changes with therapy and may distinguish viable hypoxic areas from nonviable necrotic areas in tumors [1–3]. Several PET radiotracers that target hypoxia have been developed and evaluated in research and clinical settings. Oxygen-sensitive fluorinated-nitroimidazoles are common hypoxia PET radiotracers, and the characteristics of these radiotracers have been studied extensively in several clinical and preclinical studies [2, 4]. Among the 2-nitromidazole radiotracers, [18F]fluoromisonidazole ([18F]FMISO) is the most widely used and validated PET radiotracer for imaging tumor hypoxia [5, 6]. However, its optimal acquisition time is 2–4 h after injection due to its slow uptake and clearance kinetics [1, 6]. Consequently, next-generation radiotracers have been developed with improved kinetic properties [1, 4]. Among these, [18F]fluoroazomycin-arabinoside ([18F]FAZA) is gaining acceptance for hypoxia imaging owing to its improved kinetic characteristics [1, 7, 8]. [18F]FAZA that displays a lower octanol water partition coefficient (log *P* = 1.1) is more hydrophilic than [18F]FMISO (log *P* = 2.6), increasing contrast and image quality in a shorter scan time due to a higher perfusion rate and a faster clearance from blood [6, 8, 9].

Most clinical PET studies have investigated hypoxia using late static scans. However, dynamic PET can be more informative to document the pharmacokinetics and pharmacodynamics of radiotracers that target hypoxia [8]. We designed a clinical observational study using dynamic PET/CT to evaluate the imaging characteristics of hypoxia PET tracers, [18F]FMISO and [18F]FAZA, in dogs with spontaneous tumors. The purpose of this study was to investigate the impact of scan time and duration on image contrast and consistency for tumor hypoxia detection and to assess the correlation of kinetic parameters with static imaging measures.

## Materials and Methods

### Patient Population

This was a prospective canine clinical observational study. Companion dogs were referred to the Veterinary Medical Teaching Hospital for evaluation and treatment of spontaneous tumors. Sixteen dogs were included in the study. The research protocol was approved by the Animal Care and Use Committee and Veterinary Clinical Trial Review Board, and all dog owners signed a written informed consent form. All dogs enrolled for this study had at least one spontaneous tumor in the head, neck, body wall, or limb. Inclusion criteria were as follows: solid tumors with measurable tumor volume based on tissue biopsy and imaging characteristics, body weight greater than 5 kg, absence of critically concomitant systemic diseases (diabetes, liver failure, or renal failure), and no previous history of radiation therapy. Animals were randomized into blocks of two, alternating between [18F]FMISO and [18F]FAZA groups (*N* = 8 for each group).

### Imaging Procedure

Canine patients underwent either [18F]FMISO or [18F]FAZA PET/CT imaging. Dogs were fasted for at least 12 h prior to the scan, were premedicated with midazolam and butorphanol, induced with a bolus injection of propofol, and then anesthetized with isoflurane (1–2%) in 100% oxygen via an endotracheal tube. Scans were performed on a Mini-EXPLORER II (United Imaging Healthcare, Shanghai, China) PET/CT scanner [10]. A CT scan was obtained for attenuation correction and image analysis purposes (120 kVp,175 mA). Subsequently, dogs were injected intravenously with 46–300 MBq (4.7–7.9 MBq/kg) of the respective radiotracer simultaneously with the start of PET scanning. PET list mode data were acquired for a single bed position (centered over the tumor) for 150 min. Following dynamic PET scans, a contrast-enhanced CT scan was performed. All PET images were reconstructed into a 128 × 128 × 206 matrix (voxel dimensions, 2 × 2 × 2.34 mm^3^) using a 3D time-of-flight list-mode ordered-subset expectation maximization method provided by the scanner manufacturer (4 iterations, 20 subsets, and post-reconstruction non-local means filtering). Dynamic PET reconstructions consisted of the following framing: 24 × 5, 6 × 30, 5 × 300, and 12 × 600 s (total duration,150 min).

### VOI Definition and Analysis

**Fig. 1:**
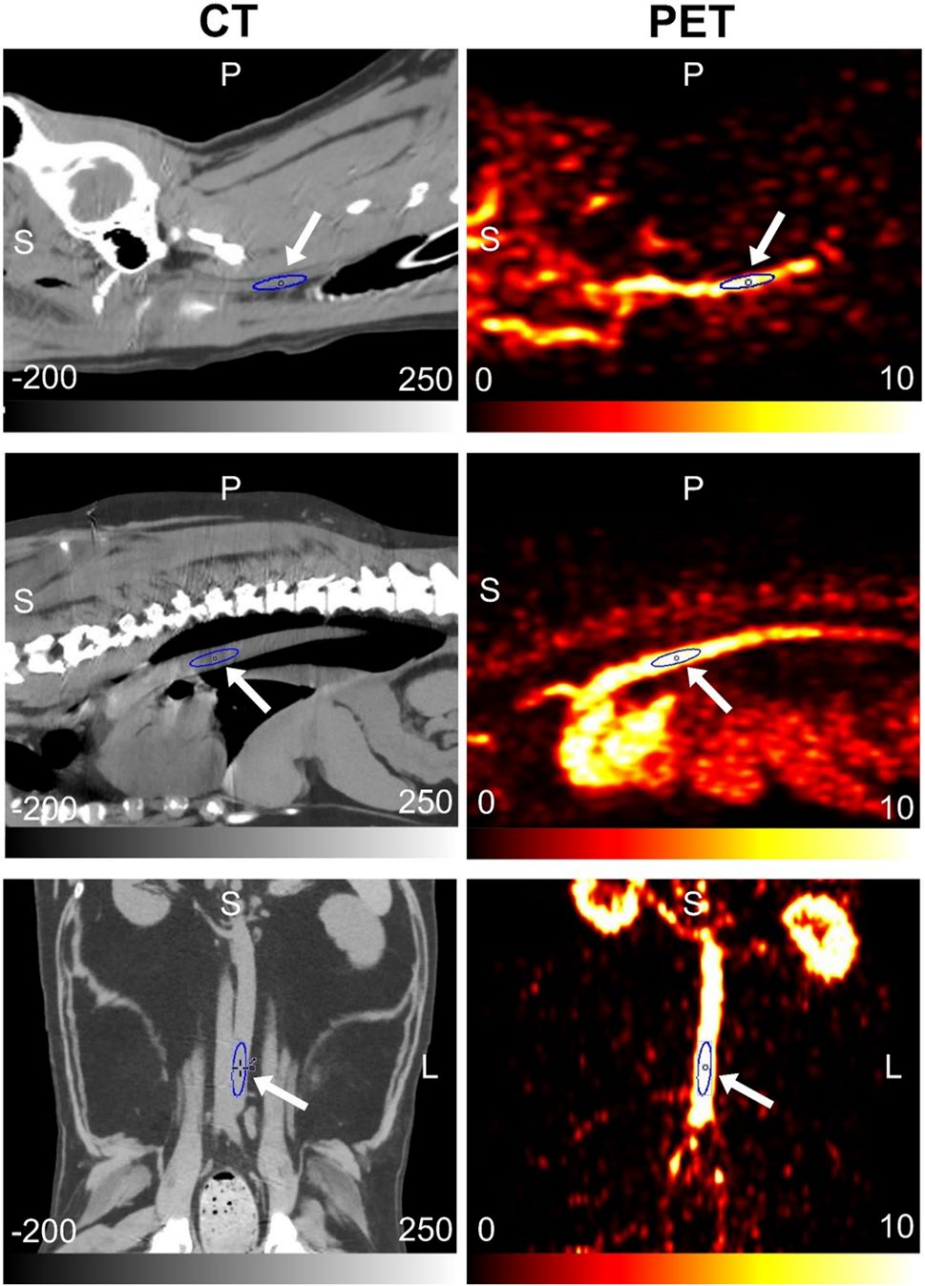
CT (left column) and PET (right column) images of carotid artery VOI (top row), thoracic aorta VOI (middle row), and abdominal aorta VOI (bottom low). Arrows indicate each artery VOI

All post-imaging analyses were performed using PMOD, version 4.1 (PMOD Technologies Ltd., Zürich, Switzerland). To extract the image-derived input function (IF), spheroid volumes-of-interest (VOIs) were defined manually on the dynamic PET frame showing the most conspicuous blood pool. Each VOI was selected at a site of a specific blood vessel as follows: the carotid artery for head and neck tumors, descending aorta for thoracic wall tumors, and abdominal aorta for flank and pelvic limb tumors (Fig. 1). The shorter and longer diameters of VOIs were at least greater than 5 and 20 mm, respectively (median volume: 0.36 cm^3^, range: 0.32–0.84 cm^3^). A fused PET/CT images were used to ascertain that the VOIs were well within the edges of the arterial wall to reduce the partial-volume effects.

Tumor VOIs were drawn manually on the unenhanced CT image, and contrast CT image was used for better delineation in cases where the tumor margin was unclear. For the non-hypoxic reference tissue, spherical VOIs with radius of 10 mm were drawn in adjacent skeletal muscle depending on the tumor location: neck muscle for tumors in the head and neck, supraspinatus muscle for tumors in the thoracic wall, and gluteal muscle for tumors in the flank, anal sac, and pelvic limb. Then, the VOIs derived, VOI_tumor_ and VOI_muscle_ were superimposed on the frames of the dynamic PET image set. Accuracy of co-registration of the VOIs and absence of frame-by-frame motion was verified by visually inspecting the full scan and time-activity curve (TAC).

Standard uptake values (SUVs) and tumor-to-muscle ratios (TMR) were calculated. TMR_max_ > 1.4 on the last image frame was used as the standard threshold to determine tumor hypoxia. In addition, hypoxic volume (HV) was defined as the volume containing voxels with TMR > 1.4, and a tumor fractional HV (FHV) was calculated as follows: FHV (%) = 100 × HV/Tumor volume.

To evaluate the consistency of HV, PET images of 10-min frames ending at 60, 90, 120, and 150 min after injection were compared to images of the last frame (150 min). The change of HV was calculated as follows: change of HV (%) = 100 × HV_(t)_/HV_150_ (HV_(t)_: HV at acquisition time *t* after injection).

### Radiotracer Kinetic Modeling

Kinetic modeling was performed using the mean TAC from each VOI (blood vessel, tumor, HV, and muscle). The irreversible two-tissue compartment model (2C3K) and reversible two-tissue compartment model (2C4K) were employed, each with blood volume parameter (*v*_b_). Fitting was performed using Marquardt–Levenberg weighted least-squares optimization. The model goodness of fit was evaluated based on Akaike criterion (AIC), where the low AIC value indicate the appropriateness of the model to describe the TAC with minimal free parameters. For the 2C3K, *k*_4_ was set to 0, reflecting irreversible trapping of radiotracer and the analysis was focused on the kinetic rate constants *K*_1_, a parameter for perfusion; *k*_3_, a parameter for hypoxia-mediated entrapment; and *K*_i_, a parameter for net influx rate. For the 2C4K, *V*_T_, the volume of distribution volume was measured. In addition, Patlak and Logan analyses were performed on whole tumor TAC to determine *K*_i_ and *V*_T_, respectively. For each radiotracer, the AIC and kinetic parameters results were compared among the whole tumor, HV, and muscle. On whole tumors, *K*_1_ was compared with *k*_3_ for each radiotracer. For evaluating the correlation between SUV measures from static imaging and the kinetic parameters, TMR_max_ of the 10-min frames ending at 60, 90, 120, and 150 min were compared with *k*_3_, *K*_i_, and *V*_T_.

### Statistical Analysis

Data were analyzed using SPSS, version 25 (IBM Corp, NY, USA). The comparison of AIC between 2C3K and 2C4K was performed with the Wilcoxon signed-rank test. The Spearman’s correlation coefficient, rho, was used as the index for the correlation. The value of this coefficient ranges from − 1.0 to 1.0, with a strong correlation defined as rho > 0.7, a weak one as rho < 0.3, and values in between as moderate. *P*-values < 0.05 were considered statistically significant for all analyses.

## Results

### Patient and Tumor Characteristics

**Fig. 2:**
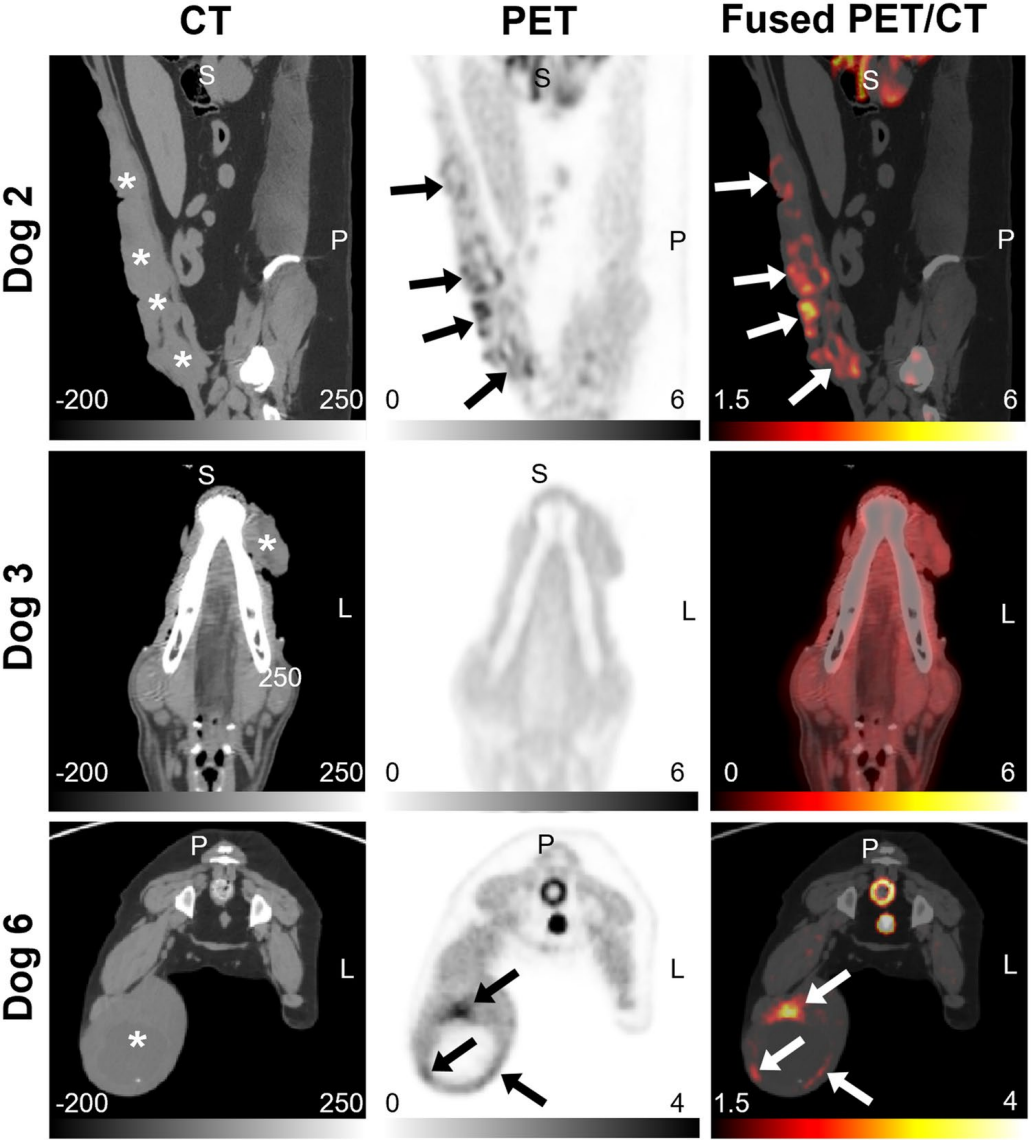
CT (left column), PET (middle column), and fused PET/CT (right column) images of canine spontaneous tumors in the [18F]FMISO group. Dog 2 (top row): carcinoma (hypoxic) in mammary gland. Dog 3 (middle row): melanoma (normoxic) in tongue. Dog 6 (bottom row): soft tissue sarcoma (hypoxic) in pelvic limb. Stars and arrows indicate tumor locations and hypoxic areas, respectively

**Fig. 3:**
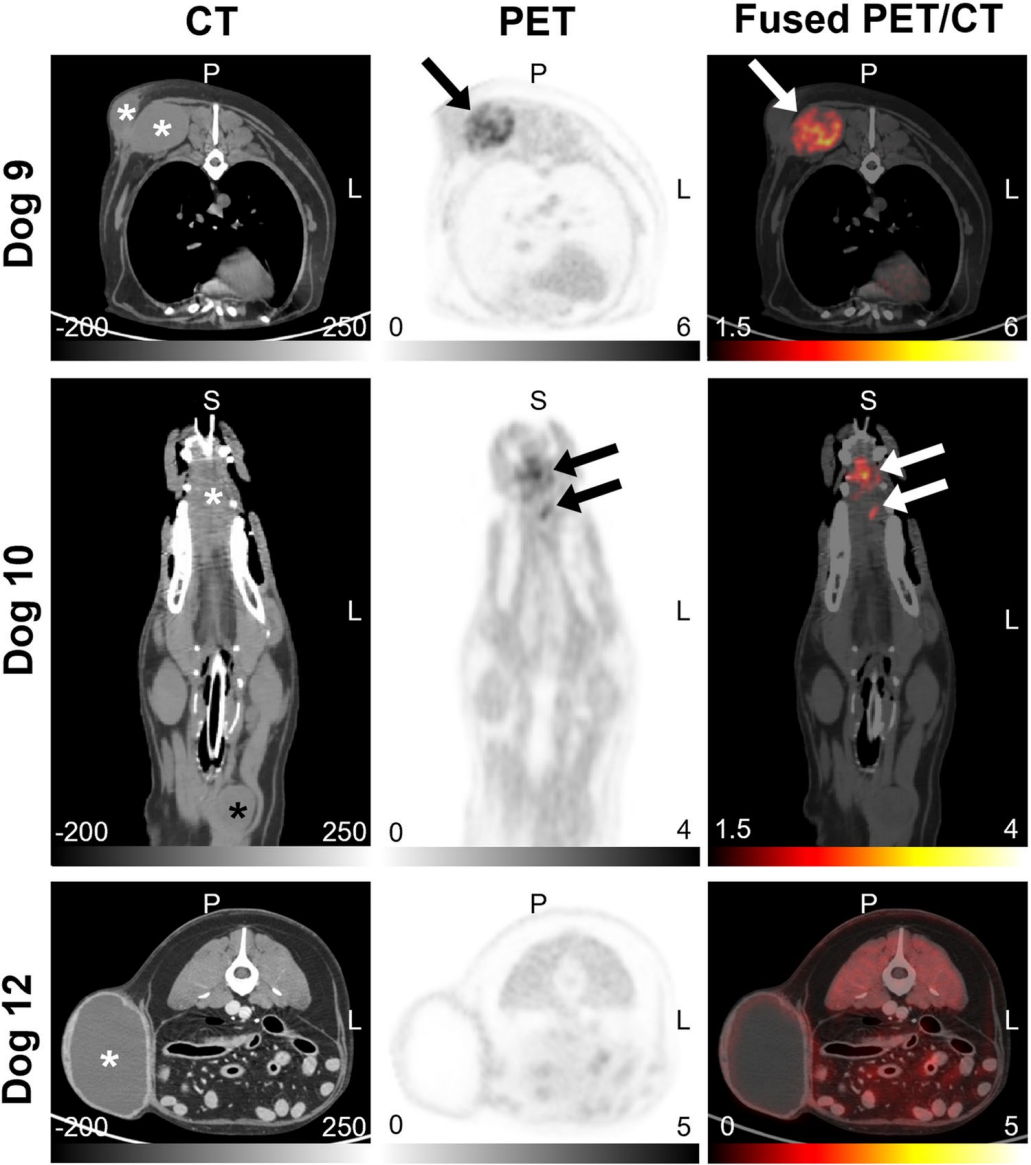
CT (left column), PET (middle column), and fused PET/CT (right column) images of canine spontaneous tumors in [18F]FAZA group. Dog 9 (top row): fibrosarcoma (hypoxic) in thoracic wall. Dog 10 (middle row): squamous cell carcinoma (hypoxic) in tongue (white star) and adenocarcinoma (normoxic) in thyroid gland (black star). Dog 12 (bottoom low): soft tissue sarcoma (normoxic) with central necrosis in flank. Stars and arrows indicate tumor locations and hypoxic areas, respectively

A total of 16 dogs were enrolled. Dog 10 in the [18F]FAZA group had two tumors. Table 1 provides details about the canine patients, including the tumor location, histopathologic diagnosis, and tumor imaging characteristics (SUV_max_ and TMR_max_ from the last-time-point images, tumor volume from CT images, and FHV). The mean tumor volumes were 120.06 cm^3^ for the [18F]FMISO group and 152.26 cm^3^ for the [18F]FAZA group. Dog 16 was excluded from analysis due to a peribladder artifact that confounded tumor assessment. Tumor hypoxia was detected in 7/8 tumors in the [18F]FMISO group and 4/8 tumors in the [18F]FAZA group. Example images of hypoxic and normoxic tumors (TMR_max_ < 1.4 on the last image frame) with each radiotracer are shown in Figs. 2 and 3.

**Table 1:** Tumor characteristics and [18F]FMISO and [18F]FAZA PET imaging results in patients

Group	Dog # (Lesion #)	Tumor location	Histology	SUV_max_	TMR_max_	Tumor Volume (cm^3^)	FHV (%)
[18F]FMISO (*n* = 8)	1 (1)	Oral cavity	Melanoma	3.37	1.80	25.46	4.36
	2 (2)	Mammary gland	Carcinoma	5.36	3.96	222.91	27.42
	3 (3)	Tongue	Melanoma	1.87	1.39	2.96	0
	4 (4)	Maxilla	Fibrosarcoma	2.72	2.12	17.92	1.73
	5 (5)	Oral cavity	Melanoma	3.60	2.61	18.88	9.64
	6 (6)	Pelvic limb	Soft tissue sarcoma	3.71	2.92	588.45	6.60
	7 (7)	Salivary gland	Carcinoma	1.90	3.11	44.51	27.77
	8 (8)	Thoracic wall	Soft tissue sarcoma	3.12	2.06	39.39	5.20
	9 (9)	Thoracic wall	Fibrosarcoma	5.38	3.28	411.09	15.50
[18F]FAZA (*n* = 8)	10 (10)	Tongue	Squamous cell carcinoma	2.92	2.78	17.76	34.74
	10 (11)	Thyroid gland	Adenocarcinoma	1.23	1.17	17.34	0
	11 (12)	Anal sac	Carcinoma	1.04	1.10	31.01	0
	12 (13)	Flank	Soft tissue sarcoma	1.06	1.03	445.06	0
	13 (14)	Nasal cavity	Chondrosarcoma	2.39	2.53	132.12	1.21
	14 (15)	Pelvic limb	Osteosarcoma	3.17	2.84	156.24	5.15
	15 (16)	Thoracic wall	Soft tissue sarcoma	0.96	1.08	47.15	0
	16 (17)	Flank	Soft tissue sarcoma	N/A	N/A	112.56	N/A

### Radiotracer Distribution and Static PET Analyses

**Fig. 4:**
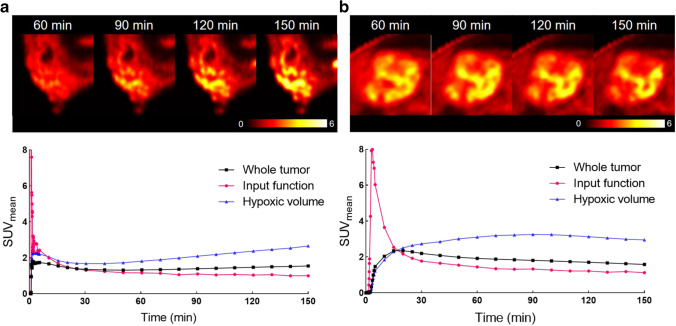
Representative PET images of canine spontaneous hypoxic tumors (top) obtained in the 60-, 90-, 120-, and 150-min timeframes after radiotracer injection and time-activity curves (bottom). **a** [18F]FMISO PET scan of mammary gland carcinoma (dog 2). **b** [18F]FAZA PET scan of fibrosarcoma in thoracic wall (dog 9). [18F]FAZA showed acceptable consistency of hypoxic volume between 120- and 150-min timeframes

Figure 4 shows representative PET images for both radiotracers in the 10-min time frames ending at 60, 90, 120, and 150 min and the mean TAC of the image-derived IF, hypoxic tumor, and HV. Hypoxic tumors showed increasing SUV_max_ over time with [18F]FMISO, whereas with [18F]FAZA, they showed a plateau phase for SUV_max_ after 120 min (Figure S1a: see ESM). Muscular uptake showed a similar pattern for both radiotracers, with the highest uptake at 60 min, decreasing from 60 to 150 min (Figure S1b: see ESM). For both tracers, contrast continues to increase over time (Figure S1c: see ESM). In each group, the presence of tumor hypoxia (TMR_max_ > 1.4) was detected in all hypoxic tumors from 120 min onward for [18F]FMISO and from 60 min onwards for [18F]FAZA. HV obtained in the 10-min frames ending at 60, 90, and 120 min was compared to those from the 10-min frame ending at 150 min (Table S1 and Fig. 4: top: see ESM). Assuming the change threshold for HV less than 10% is adequate for reproducibility, [18F]FAZA images met this threshold at 120 min in most of hypoxic tumors (3/4), compared with 2/7 hypoxic tumors in [18F]FMISO images.

### Radiotracer Kinetics Analysis

The AIC results are summarized in Table 2. Theoretically, the uptake mechanism of nitroimidazole would indicate an irreversible model. Therefore, for quantification, 2C3K has been generally used for [18F]FMISO PET studies [3, 8, 11, 12]. In this study, for [18F]FMISO, the AIC measured for HV was not significantly different between the 2C3K and 2C4K. The kinetic parameters (*k*_3_ and *K*_i_) derived from 2C3K, however, showed a higher correlation (rho: 0.76 in *k*_3_-TMR_max_ and 0.74 in *K*_i_-TMR_max_, *p* < 0.05) with tumor hypoxia evaluated from late-stage PET uptake. On the contrary, for [18F]FAZA, the TACs of all VOIs showed better fits with a 2C4K. Therefore, the remainder of this study focused on [18F]FMISO kinetic analysis using the 2C3K and Patlak plot and [18F]FAZA kinetic analysis using the 2C4K and Logan plot.

**Table 2:** Comparison of AIC values between 2C3K and 2C4K for [18F]FMISO and [18F]FAZA

	AIC							
	[18F]FMISO				[18F]FAZA			
	*n*	2C3K	2C4K	*p*-value	*n*	2C3K	2C4K	*p*-value
Tumor	8	39.85 (3.07–78.00)	22.03 (1.94–44.75)	< 0.05	8	75.74 (43.66–124.99)	29.85 (4.81–54.78)	< 0.05
Hypoxic volume	7	29.68 (4.12–99.53)	32.76 (6.01–94.45)	0.612	4	62.21 (31.78–125.58)	44.45 (16.49–81.09)	0.068
Muscle	8	65 (7.19–122.27)	24.03 (5.6–124.23)	0.263	7	98.84 (18.76–159.14)	77.11 (17.82–138)	< 0.05

Data are presented as median (range)

**Fig. 5:**
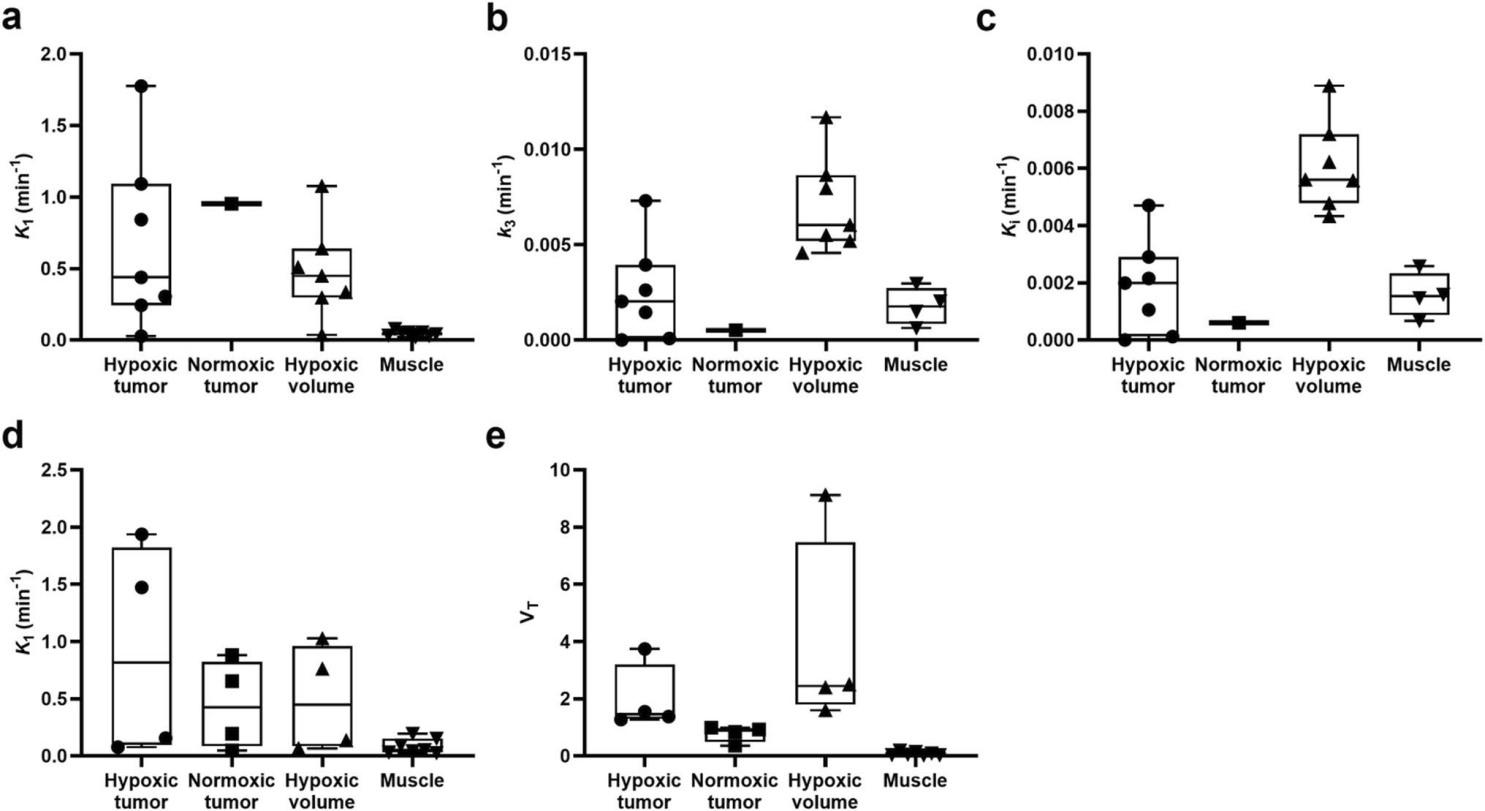
Comparison between kinetic parameters determined for the averaged time-activity curves of the 4 VOIs (VOI_hypoxic tumor_, VOI_normoxic tumor_, VOI_hypoxic volume_, and VOI_muscle_) in each kinetic model: 2C3K parameters; **a** *K*_1_, **b** *k*_3_, and **c** *K*_i_ in [18F]FMISO and 2C4K parameters; **d** *K*_1_ and **e** V_T_ in [18F]FAZA. A significant number of [18F]FMISO hypoxic tumors (5/7) showed higher *k*_3_ and *K*_i_ than the normoxic tumor (*n* = 1). [18F]FAZA showed that all hypoxic tumors (*n* = 4) exhibited greater *V*_T_ than the normoxic tumors (*n* = 4). In each group, all HVs showed greater kinetic parameter values (*k*_3_ and *K*_i_ for [18F]FMISO and *V*_T_ for [18F]FAZA), compared to hypoxic tumors. The boundaries of the boxes closest to zero represent the 25th percentiles, lines in the boxes indicate the medians, boundaries of boxes farthest from zero represent the 75th percentiles, and the whiskers correspond to min and max values

Kinetic modeling results for all VOIs are summarized and compared (Fig. 5; Table S2; see ESM). A significant number of [18F]FMISO hypoxic tumors (5/7) showed higher 2C3K *k*_3_ and *K*_i_ (> 0.001) than the normoxic tumor except for 2/7 hypoxic tumors (2C3K *k*_3_ and *K*_i_ < 0.0002 min^−1^) containing the least FHV. In addition, most hypoxic tumors (6/7) showed Patlak *K*_i_ > 0 except for the hypoxic tumor containing least FHV (1.73%), but Patlak *K*_i_ < 0 was observed in the normoxic tumor. [18F]FAZA showed that hypoxic tumors exhibited significantly greater 2C3K and Logan *V*_T_ (> 1.26) than the normoxic tumors (*p* < 0.05, Mann–Whitney test). In each group, all HVs showed greater kinetic parameter values (2C3K *k*_3_ and *K*_i_ for [18F]FMISO (*p* < 0.05, Wilcoxon signed-rank test) and 2C4K *V*_T_ for [18F]FAZA), compared to hypoxic tumors. Greater values (2C3K *k*_3_ and *K*_i_ > 0.004) were also presented for HVs of two hypoxic tumors having the least FHV. Interestingly, for [18F]FMISO, strong negative correlation was found between *K*_1_ and *k*_3_ (rho = − 0.76, *p* = 0.014).

### Comparison of Static and Dynamic PET Measures

When kinetic parameters were compared to simplified quantification measures (TMR_max_) from static imaging obtained in 10-min frames ending at 60, 90, 120, and 150 min, a strong positive correlation was found in 2C3K *k*_3_-TMR_max_ and 2C3K *K*_i_-TMR_max_ for the frames ending at 90, 120, and 150 min for [18F]FMISO and between 2C4K *V*_T_ and TMR_max_ for frames ending at 120 and 150 min for [18F]FAZA (Table 3). Graphical analyses showed a strong positive correlation between Patlak *K*_i_ and TMR_max_ at 90 min for [18F]FMISO and Logan *V*_T_ and TMR_max_ at 120- and 150-min frames for [18F]FAZA. [18F]FMISO 2C3K *k*_3_ and *K*_i_ showed the strongest correlation with TMR_max_ (rho = 0.83, *p* = 0.005, for both) in the frames ending at 90 and 120 min, respectively. For [18F]FAZA PET, the strongest correlation in *V*_T_-TMR_max_ was identified from the 10-min frame ending at 150 min (2C3K *V*_T_-TMR_max_: rho = 0.86, *p* = 0.003, Logan *V*_T_-TMR_max_: rho = 0.83, *p* = 0.005), with increasing rho over time.

**Table 3:** Correlation results between hypoxic parameters (*k*_3_ and *K*_i_ for [18F]FMISO and V_T_ for [18F]FAZA) from kinetic and graphical modeling versus TMR_max_ of static imaging for 10-min timeframes ending at 60, 90, 120, and 150 min

Time (min)	[18F]FMISO						[18F]FAZA			
	*k*_3_ (2C3K) vs. TMR_max_		*K*_i_ (2C3K) vs. TMR_max_		*K*_i_ (Patlak) vs. TMR_max_		*V*_T_ (2C4K) vs. TMR_max_		*V*_T_ (Logan) vs. TMR_max_	
	Rho	*p*-value	Rho	*p*-value	Rho	*p*-value	Rho	*p*-value	Rho	*p*-value
60	0.45	0.13	0.52	0.091	0.33	0.21	0.57	0.069	0.62	0.051
90	0.83	0.005	0.81	0.007	0.76	0.014	0.67	0.035	0.69	0.025
120	0.81	0.007	0.83	0.005	0.67	0.035	0.83	0.005	0.81	0.007
150	0.76	0.014	0.74	0.017	0.69	0.029	0.86	0.003	0.83	0.005

## Discussion

Over the years, several fluorinated nitroimidazole-based PET radiotracers have been developed for tumor hypoxia imaging. Among all the radiotracers in this group, [18F]FMISO and [18F]FAZA are the most promising hypoxia-specific radiotracers [7]. Kinetic analysis of these tracers is a critical consideration for assessing the underlying physiology behind tracer uptake in molecular imaging [13, 14]. Previous studies have compared the radiotracer kinetics of [18F]FAZA with [18F]FMISO and have demonstrated an improved contrast at earlier time points in [18F]FAZA [8]. However, radiotracer uptake patterns may differ between implanted tumors in rodents, relative to the spontaneous tumors that develop over time in human and dog patients. Moreover, most commonly used mouse tumor models of implanted subcutaneous tumors develop different architecture, vasculature, and hypoxia patterns than spontaneously occurring orthotopic tumors, limiting their translational value in hypoxia imaging studies. Arguably, compared to inbred and genetically modified laboratory animals, dogs with spontaneous neoplasms better reflect biology, anatomy, and therapeutic responses of human tumors as well as share with humans similar environmental epigenetics [15]. Therefore, this study using dogs affected with spontaneous cancer could inform human clinical studies and trials aiming to further develop more translatable protocols for assessing tumor hypoxia. To our best knowledge, this is first study to evaluate the kinetics of [18F]FAZA using dynamic PET/CT in canine spontaneous tumors.

Most clinical studies in hypoxia imaging with PET have suggested static scan times beginning 2–4 h after [18F]FMISO and [18F]FAZA administration [1, 16]. Therefore, we performed dynamic PET scan within that time (150 min) to determine the radiotracer uptake characteristics in hypoxic tumors. As clinically demonstrated, while [18F]FMISO did not show a plateau formation and increased tracer uptake in hypoxic tumors at later time point, [18F]FAZA had a plateau phase for tracer uptake after 120 min [2]. Due to its faster uptake and clearance kinetics, the presence of tumor hypoxia (TMR_max_ > 1.4) was detected in all hypoxic tumors from 120 min onward for [18F]FMISO and from 60 min onwards for [18F]FAZA.

Validation of the consistency of radiotracer uptake is a vital prerequisite to clinical applications (e.g., anti-hypoxic drugs and intensity-modulated radiotherapy). It would be desirable to have a radiotracer that shows high consistency over time in a longitudinal or interventional study. In our results, [18F]FAZA showed the plateau phase of SUV_max_ after 120 min and a higher rate of HV consistency between 120-min and 150-min frame than [18F]FMISO. In the previous study, high consistency was found when later time images of [18F]FMISO (acquired at 6 h) were compared to images acquired at 2, 3, 4, and 5 h [2]. Other studies have reported that due its slow kinetics, [18F]FMISO PET imaging at 4 h after injection was considered more reproducible compared with imaging at less than 3 h post injection [17, 18]. However, these longer wait times lead to lower signal-to-noise ratio due to radioactive F-18 decay, and long dynamic image acquisition was deemed too great a burden for the anesthetized canine patients in this study.

In the present study, kinetics of [18F]FMISO and [18F]FAZA were investigated using both compartment and graphical models. Under hypoxic conditions, nitroimidazoles are reduced and mostly become irreversibly bound to macromolecules in the cell at rates that are inversely proportional to the local pO_2_ [1, 9]. Therefore, for [18F]FMISO, 2C3K is generally used for kinetic analysis. Our results were consistent with these data. However, in early timepoint analysis of [18F]FMISO uptake, several tumoral TACs appeared to be consistent with the 2C4K model. This was attributed to the large heterogeneity of these tumors and non-specific binding early in the uptake phase [19]. For [18F]FAZA, lower AIC in all VOIs was identified in the 2C4K, demonstrating that the radiotracer was reversibly bound to tumor. This finding is consistent with results of [18F]FAZA in human tumors of lung, head, and neck [14, 20]. This result might be due to a lower octanol/water partition coefficient of [18F]FAZA, leading to more rapid diffusion thorough tissue and faster renal excretion [19].

The kinetic analysis results in this study showed that whereas each surrogate parameter of hypoxia for [18F]FMSIO and [18F]FAZA was generally greater in hypoxic tumors versus non-hypoxic tumors, [18F]FMISO 2C3K *k*_3_ and *K*_i_ were less than 0.001 min^−1^ in 2/7 hypoxic tumors having the least FHV. This is likely caused by the large fraction of normoxic volume compared to HV that could make mean TAC_tumor_ more dependent on the well perfused-normoxic regions [21], leading to an underestimation of tumor hypoxia. When kinetic analyses were performed using HV, greater values were found in these two tumors. This indicates that voxel-based data analysis can more precisely reflects the radiotracer behavior in smaller subvolumes within the tumor [8]. For [18F]FMISO, strong negative correlation was found between perfusion and tumor hypoxia represented by *K*_1_ and *k*_3_, respectively. This finding supports the hypothesis that limitation in oxygen diffusion causes development of hypoxic regions far from blood vessels, leading to diffusion-limited hypoxia [3]. However, this result has to be confirmed in larger cohorts.

The most widely used simplified quantification of tumor hypoxia in PET imaging is TMR_max_ derived from a single late-time static image. Although a threshold for the definition of hypoxia has not been formally established, several clinical studies in [18F]FMISO and [18F]FAZA have reported that TMR > 1.4 imaged after at least 2 h post injection can be generally considered as indicative of hypoxia [1, 16]. However, the possibility of a cross-over point between the decline in activity from normoxic tissue and the increasing activity from a hypoxic tissue can result in an ambiguity in the interpretation of single-time-point imaging [3, 20]. Therefore, for validation of simplified quantification of static imaging, we investigated correlations between kinetic parameters of hypoxia and TMR_max_ at several time-points. Strong correlation was found in 2C3K *k*_3_-TMR_max_ and 2C3K *K*_i_-TMR_max_ from 90 min onwards for [18F]FMISO and in 2C4K and Logan *V*_T_-TMR_max_ from 120 min for [18F]FAZA, indicating that static imaging from these timepoints is a suitable alternative to full kinetic modeling for quantifying radiotracer uptake.

When evaluating PET/CT images, radiotracer accumulation in the urinary bladder should be considered. Dog 16 was excluded from analysis due to the peribladder artifact. In a study of rectal cancer, the scattered activity of [18F]FAZA from the urinary bladder complicated the interpretation of tumor hypoxia [22]. Because voluntary voiding is impossible in anesthetized animals, this artifact could be minimized by urinary catheterization or cystocentesis before the PET/CT scan.

The major limitations of this prospective randomized clinical trial were that only eight patients were enrolled for the kinetic evaluation of tumor hypoxia in each [18F]FMISO and [18F]FAZA group, and a variety of spontaneous tumors were included, rather than restricted to a specific type of lesion, which resulted in the failure to compare and detect a statistically significant difference between the behavior of the two tracers. This study did not include independent and invasive techniques such as oxygen electrodes or immunohistochemical staining. While oxygen electrodes are often considered the gold standard for tumor hypoxia measurement, their application is restricted by user dependence and sampling error [3, 16]. Several studies correlating direct oxygen measurement with tracer accumulation in vivo have shown mixed and contradictory results [1], which was attributed to the variable sampling location of representative tumor biopsies and technically challenging spatial co-registration between PET images and immunohistochemistry staining photographs for analogies to be drawn [1, 16]. The invasiveness of these procedures limits their use to measure change in tumor hypoxia in this veterinary clinical trial using client-owned dogs. Finally, discrepancy between clinical and pre-clinical dog studies was the use of anesthetic agent and different oxygen concentration, which could have a substantial influence on the kinetics of radiotracers and tissue oxygenation. In present study, dog patients were maintained under anesthesia using 1–2% isoflurane in 100% oxygen. In CaNT-bearing CBA mice, [18F]FMISO TMR was reduced when either 100% oxygen or anesthetics were introduced [23]. Another study has reported that CT26 colorectal carcinoma bearing mice showed higher [18F]FAZA TMR when the mice breathed air compared to the 100% oxygen breathing protocols [24]. Further studies are needed to clarify whether the anesthetic protocol performed in this dog study influence the imaging of tumor hypoxia.

## Conclusion

The results of this study confirmed that [18F]FMISO and [18F]FAZA were able to detect the presence of tumor hypoxia in a variety of spontaneous canine tumors. [18F]FAZA provided a promising alternative radiotracer to [18F]FMISO with earlier detection of tumor hypoxia (60 min) and good HV consistency (120 min), consistent with its faster kinetics. In both tracers, evidence for tumor hypoxia as depicted by increased radiotracer uptake was confirmed by increased *k*_3_, *K*_i_ for [18F]FMISO and *V*_T_ for [18F]FAZA, compared to that in normoxic tumor. [18F]FMISO and [18F]FAZA showed strong correlation between simplified quantification of static imaging and kinetic results at 90–150 and 120–150 min, respectively. This indicates that TMR_max_ at these timepoints provides an alternative static PET-based measure to quantify tumor hypoxia that is comparable to the kinetic parameters.

## Supplementary Information

Below is the link to the electronic supplementary material.

Supplementary file1 (DOCX 323 kb)

## Data Availability

Contact the corresponding author for data requests*.*

## References

[1] Fleming IN, Manavaki R, Blower PJ, West C, Williams KJ, Harris AL (2015). Imaging tumour hypoxia with positron emission tomography. Br J Cancer.

[2] Peeters SGJA, Zegers CML, Lieuwes NG, Van Elmpt W, Eriksson J, Van Dongen GAMS (2015). A comparative study of the hypoxia PET tracers [18F]HX4, [18F]FAZA, and [18F]FMISO in a preclinical tumor model. Int J Radiat Oncol Biol Phys.

[3] Wang W, Lee NY, Georgi JC, Narayanan M, Guillem J, Scḧoder H (2010). Pharmacokinetic analysis of hypoxia 18F-fluoromisonidazole dynamic PET in head and neck cancer. J Nucl Med.

[4] Zschaeck S, Löck S, Hofheinz F, Zips D, Saksø Mortensen L, Zöphel K (2020). Individual patient data meta-analysis of FMISO and FAZA hypoxia PET scans from head and neck cancer patients undergoing definitive radio-chemotherapy. Radiother Oncol.

[5] Quartuccio N, Laudicella R, Mapelli P, Guglielmo P, Pizzuto DA, Boero M (2020). Hypoxia PET imaging beyond 18F-FMISO in patients with high-grade glioma: 18F-FAZA and other hypoxia radiotracers. Clin Transl Imaging.

[6] Lopci E, Grassi I, Chiti A, Nanni C, Cicoria G, Toschi L (2014). PET radiopharmaceuticals for imaging of tumor hypoxia: a review of the evidence. Am J Nucl Med Mol Imaging.

[7] Mapelli P, Picchio M (2020). 18F-FAZA PET imaging in tumor hypoxia: a focus on high-grade glioma. Int J Biol Markers.

[8] Li F, Joergensen JT, Hansen AE, Kjaer A (2014). Kinetic modeling in PET imaging of hypoxia. Am J Nucl Med Mol Imaging.

[9] Morand P, Hans-Jürgen M, Maria P, Gerald R, Sybille Z, Piyush K (2005). Hypoxia-specific tumor imaging with 18F-fluoroazomycin arabinoside. J Nucl Med.

[10] Lv Y, Lv X, Liu W, Judenhofer MS, Zwingenberger A, Wisner E (2019). Mini EXPLORER II: a prototype high-sensitivity PET/CT scanner for companion animal whole body and human brain scanning. Phys Med Biol.

[11] Schwartz J, Grkovski M, Rimner A, Schöder H, Zanzonico PB, Carlin SD (2017). Pharmacokinetic analysis of dynamic 18F-fluoromisonidazole PET data in non-small cell lung cancer. J Nucl Med.

[12] Grkovski M, Schöder H, Lee NY, Carlin SD, Beattie BJ, Riaz N (2017). Multiparametric imaging of tumor hypoxia and perfusion with 18F-fluoromisonidazole dynamic PET in head and neck cancer. J Nucl Med.

[13] Reischl G, Dorow DS, Cullinane C, Katsifis A, Roselt P, Binns D (2007). Imaging of tumor hypoxia with [124I]IAZA in comparison with [18F]FMISO and [18F]FAZA - first small animal PET results. J Pharm Pharm Sci.

[14] Shi K, Souvatzoglou M, Astner ST, Vaupel P, Nüsslin F, Wilkens JJ (2010). Quantitative assessment of hypoxia kinetic models by a cross-study of dynamic 18F-FAZA and 15O–H2O in patients with head and neck tumors. J Nucl Med.

[15] Paoloni M, Khanna C (2008). Translation of new cancer treatments from pet dogs to humans. Nat Rev Cancer.

[16] Kelada OJ, Carlson DJ (2014). Molecular imaging of tumor hypoxia with positron emission tomography. Radiat Res.

[17] Okamoto S, Shiga T, Yasuda K, Ito YM, Magota K, Kasai K (2013). High reproducibility of tumor hypoxia evaluated by 18F-fluoromisonidazole pet for head and neck cancer. J Nucl Med.

[18] Nehmeh SA, Lee NY, Schröder H, Squire O, Zanzonico PB, Erdi YE (2008). Reproducibility of intratumor distribution of 18f-fluoromisonidazole in head and neck cancer. Int J Radiat Oncol Biol Phys.

[19] Verwer EE, Zegers CML, van Elmpt W, Wierts R, Windhorst AD, Mottaghy FM (2016). Pharmacokinetic modeling of a novel hypoxia PET tracer [18F]HX4 in patients with non-small cell lung cancer. EJNMMI Phys.

[20] Verwer EE, Van Velden FHP, Bahce I, Yaqub M, Schuit RC, Windhorst AD (2013). Pharmacokinetic analysis of [18F]FAZA in non-small cell lung cancer patients. Eur J Nucl Med Mol Imaging.

[21] Thorwarth D, Eschmann SM, Scheiderbauer J, Paulsen F, Alber M (2005). Kinetic analysis of dynamic 18F-fluoromisonidazole PET correlates with radiation treatment outcome in head-and-neck cancer. BMC Cancer.

[22] Havelund BM, Holdgaard PC, Rafaelsen SR, Mortensen LS, Theil J, Bender D (2013). Tumour hypoxia imaging with 18F-fluoroazomycinarabinofuranoside PET/CT in patients with locally advanced rectal cancer. Nucl Med Commun.

[23] Kersemans V, Cornelissen B, Hueting R, Tredwell M, Hussien K, Allen PD (2011). Hypoxia imaging using PET and SPECT: The effects of anesthetic and carrier gas on [64cu]-ATSM, [99mTc]-HL91 and [18F]-fmiso tumor hypoxia accumulation. PLoS ONE.

[24] Maier FC, Kneilling M, Reischl G, Cay F, Bukala D, Schmid A (2011). Significant impact of different oxygen breathing conditions on noninvasive in vivo tumor-hypoxia imaging using [18F]-fluoro-azomycinarabino-furanoside ([18F]FAZA). Radiat Oncol.

