# Supplementary material for: Kinetic Evaluation of the Hypoxia Radiotracers [18F]FMISO and [18F]FAZA in Dogs with Spontaneous Tumors Using Dynamic PET/CT Imaging
Source: Nucl Med Mol Imaging. 2022 Oct 11;57(1):16–25. doi: 10.1007/s13139-022-00780-4 (PMC9832187; doi:10.1007/s13139-022-00780-4)
Supplement: Supplementary file 1 — Supplementary file1 (DOCX 323 kb) [file 13139_2022_780_MOESM1_ESM.docx]

**Figure S1.** Time-active curves (TACs) of (a) SUV_max_ of hypoxic tumor, (b) SUV_mean_ of muscle, and (C) TMR_max_ for [18F]FMISO (n=7) and [18F]FAZA (n=4). Hypoxic tumors showed increasing SUV_max_ over time with [^18^F]FMISO, whereas with [18F]FAZA they showed a plateau phase for SUV_max_ after 120 min. Muscular uptake showed a similar pattern for both radiotracers, with the highest uptake at 60 min, decreasing from 60-150 min. For both tracers, TMR_max_ continues to increase over time. Data are means ± SD


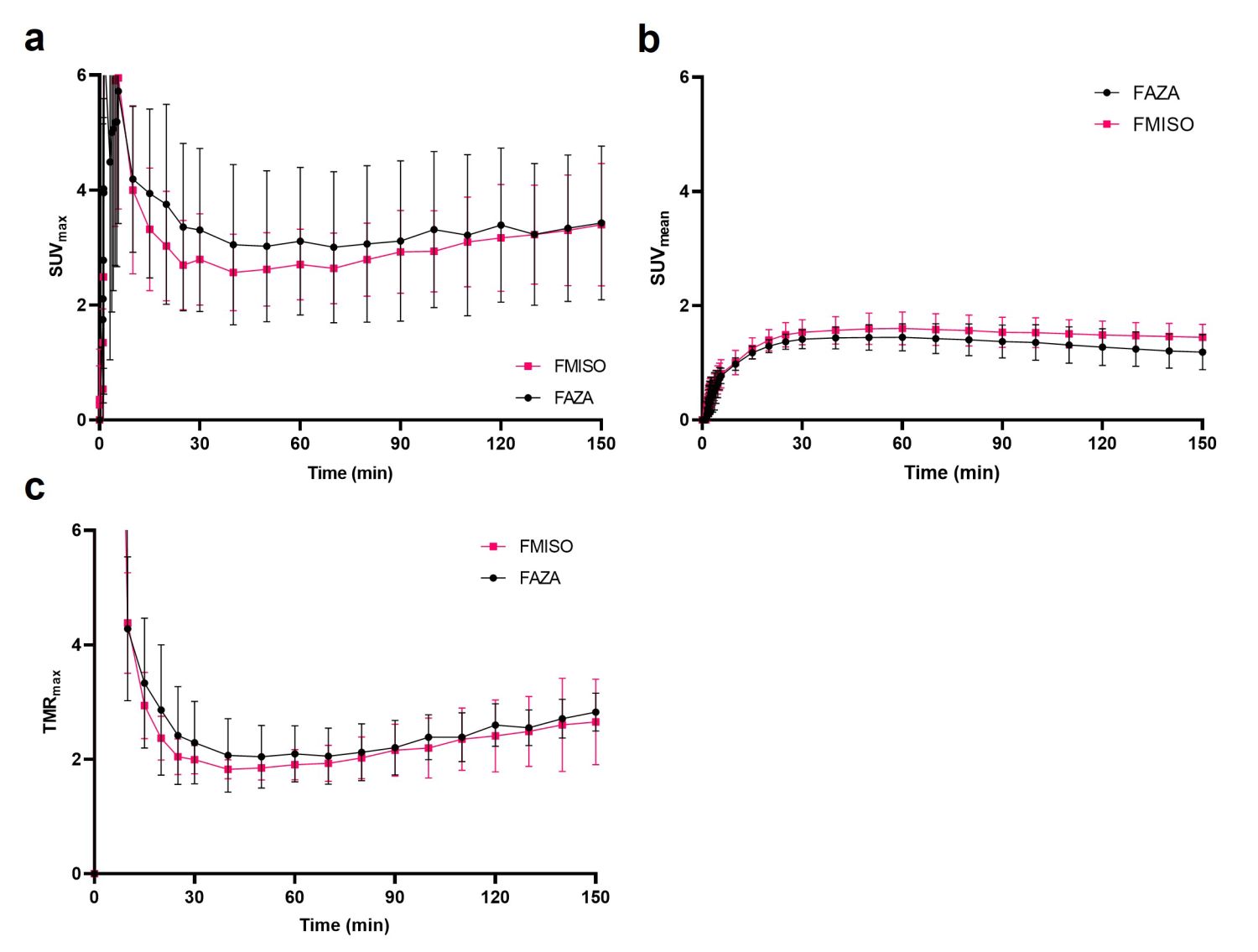


**Table S1.** The change of HV between the scan obtained for the 10-min timeframes ending at 60-, 90-, 120-min and the 150 min

|  |  | Change of HV (%) | | |
| --- | --- | --- | --- | --- |
| [18F]FMISO  (n=7) | Lesion # | 60 -150 min | 90-150 min | 120-150 min |
|  | 1 | 100 | 82.57 | 57.00 |
|  | 2 | 75.77 | 46.66 | 20.90 |
|  | 4 | 54.69 | 24.27 | 9.06 |
|  | 5 | 50.14 | 56.88 | 15.69 |
|  | 6 | 88.25 | 73.06 | 41.17 |
|  | 7 | 43.96 | 9.48 | 5.00 |
|  | 8 | 42.11 | -5.67 | 19.49 |
|  | Mean ± SD | 64.99 ± 21.30 | 41.04 ± 30.45 | 24.04 ± 17.18 |
| [18F]FAZA  (n=4) | 9 | -20.42 | -10.66 | -1.92 |
|  | 10 | -7.00 | 27.91 | 8.13 |
|  | 13 | 43.55 | 48.87 | 7.57 |
|  | 14 | 87.98 | 67.31 | 25.80 |
|  | Mean ± SD | 26.03 ± 42.99 | 33.36 ± 28.98 | 9.90 ± 10.01 |

**Table S2.** Summary of kinetic analysis results for all analyzed tissues in [18F]FMISO and [18F]FAZA. Data are summarized as median (range)

|  |  | 2C3K | | | Patlak | 2C4K | | Logan |
| --- | --- | --- | --- | --- | --- | --- | --- | --- |
|  |  | *K*_1_ (mL/min/cm^3^) | *k*_3_ (min^-1^) | *K*_i_ (min^-1^) | *K*_i_ (min^-^) | *K*_1_ (mL/min/cm^3^) | V_T_ | V_T_ |
| [18F]FMISO | Hypoxic  tumor  (n=7) | 0.438  (0.027-1.775) | 0.002  (0-0.007) | 0.002  (9.77E-10-  0.005) | 4.26E-0.4  (1.46E-4  -0.003) | 0.966  (0.030-2.919) | 1.387  (0.826-1.404) | 1.17  (0.849-1.342) |
|  | Normoxic  tumor  (n=1) | 0.953 | 0.001 | 0.001 | -4.88E-04 | 1.069 | 1.248 | 1.237 |
|  | HV  (n=7) | 0.448  (0.035-1.077) | 0.006  (0.005-0.012) | 0.006  (0.004-0.009) |  | 0.479  (0.037-1.364) | 1.881  (1.399-13.977) |  |
|  | Muscle  (n=8) | 0.047  (0.021-0.078) | 0  (3.7E-201-0.003) | 0  (3.9E-300-  0.003) |  | 0.068  (0.021-0.150) | 1.100  (0.899-1.254) |  |
| [18F]FAZA | Hypoxic  tumor  (n=4) | 0.386  (0.060-0.762) | 0.006  (0.002-0.018) | 0.004  (0.001-0.010) | 0.002  (-0.001  -0.008) | 0.911  (0.077-1.938) | 1.985  (1.274-3.744) | 1.462  (1.266-1.586) |
|  | Normoxic  tumor  (n=4) | 0.275  (0.042-0.504) | 0.004  (0.001-0.009) | 0.002  (0.001-0.003) | 7.1E-4  (4.2E-4  -0.001) | 0.445  (0.049-0.882) | 0.781  (0.362-0.993) | 0.887  (0.361-0.992) |
|  | HV  (n=4) | 0.398  (0.042-0.721) | 0.011  (0.001-0.025) | 0.008  (0.002-0.013) |  | 0.498  (0.068-1.027) | 3.905  (1.601-9.127) |  |
|  | Muscle  (n=7) | 0.044  (0.024-0.065) | 0.004  (0-0.014) | 0.003  (0-0.009) |  | 0.085  (0.027-0.194) | 1.527  (0.968-3.178) |  |
